# Low 25-Hydroxyvitamin D Levels on Admission to the Intensive Care Unit May Predispose COVID-19 Pneumonia Patients to a Higher 28-Day Mortality Risk: A Pilot Study on a Greek ICU Cohort

**DOI:** 10.3390/nu12123773

**Published:** 2020-12-09

**Authors:** Alice G. Vassiliou, Edison Jahaj, Maria Pratikaki, Stylianos E. Orfanos, Ioanna Dimopoulou, Anastasia Kotanidou

**Affiliations:** 1 First Department of Critical Care Medicine & Pulmonary Services, School of Medicine, National and Kapodistrian University of Athens, Evangelismos Hospital, 106 76 Athens, Greece; alvass75@gmail.com (A.G.V.); edison.jahaj@gmail.com (E.J.); sorfanos@med.uoa.gr (S.E.O.); idimo@otenet.gr (I.D.); 2 Biochemical Department, Evangelismos Hospital, 106 76 Athens, Greece; marypratik@icloud.com; 3 Second Department of Critical Care, School of Medicine, National and Kapodistrian University of Athens, Attikon Hospital, 124 62 Athens, Greece

**Keywords:** vitamin D, SARS-CoV2, ICU, outcomes, mortality

## Abstract

We aimed to examine whether low intensive care unit (ICU) admission 25-hydroxyvitamin D (25(OH)D) levels are associated with worse outcomes of COVID-19 pneumonia. This was a prospective observational study of SARS-CoV2 positive critically ill patients treated in a multidisciplinary ICU. Thirty (30) Greek patients were included, in whom 25(OH)D was measured on ICU admission. Eighty (80%) percent of patients had vitamin D deficiency, and the remaining insufficiency. Based on 25(OH)D levels, patients were stratified in two groups: higher and lower than the median value of the cohort (15.2 ng/mL). The two groups did not differ in their demographic or clinical characteristics. All patients who died within 28 days belonged to the low vitamin D group. Survival analysis showed that the low vitamin D group had a higher 28-day survival absence probability (log-rank test, *p* = 0.01). Critically ill COVID-19 patients who died in the ICU within 28 days appeared to have lower ICU admission 25(OH)D levels compared to survivors. When the cohort was divided at the median 25(OH)D value, the low vitamin D group had an increased risk of 28-day mortality. It seems plausible, therefore, that low 25(OH)D levels may predispose COVID-19 patients to an increased 28-day mortality risk.

## 1. Introduction

Vitamin D deficiency is known to aggravate the incidence and outcome of infectious complications, especially in patients admitted to the intensive care unit (ICU) [1]. Host defense against intracellular pathogens depends upon innate and adaptive antimicrobial effector pathways. One such important pathway is the Toll-like receptor (TLR) pathway, which is activated by 25-hydroxyvitamin D (25(OH)D) [2,3]. More specifically, 25(OH)D regulates the expression of the antimicrobial peptides cathelicidin and β-defensin, which may help improve endothelial barrier function [4].

The severity of coronavirus 2019 disease (COVID-19) can be manifested by the presence of pneumonia, severe acute respiratory distress syndrome, myocarditis, microvascular thrombosis and/or cytokine storms, all of which are known to involve inflammatory responses. Low 25(OH)D levels, on the contrary, have been associated with increased levels of inflammatory cytokines and an increased risk of pneumonia and viral upper respiratory tract infections [5]. To this end, clinical trials with 25(OH)D administration are being carried out in COVID-19 patients in an effort to improve outcomes [6]. 

Hence, we assumed that lower 25(OH)D levels on ICU admission could predispose COVID-19 positive patients to worse outcomes, such as increased risk of 28-day mortality. 

## 2. Materials and Methods

The study was approved by the “Evangelismos” Hospital Research Ethics Committee (129/19-3-2020), and all procedures carried out on patients were in compliance with the Helsinki Declaration. Informed written consent was obtained from all patients’ next of kin. 

This prospective, observational study included consecutive, critically ill patients of Greek ethnicity, suffering from COVID-19 pneumonia, who were directly admitted to the ICU of the “Evangelismos” General Hospital from 22 March to 3 August 2020. SARS-CoV-2 infection was diagnosed by real-time reverse transcription PCR (RT-PCR) in nasopharyngeal swabs. ICU admission 25(OH)D measurements were available for 36 patients. Finally, 30 Greek patients were enrolled. Apart from ethnicity, there were no other exclusion criteria. Following study enrolment, demographic characteristics, comorbidities, symptoms, vital signs, laboratory findings and COVID-19-targeted compounds were recorded. Acute Physiology and Chronic Health Evaluation (APACHE II) and Sequential Organ Failure Assessment (SOFA) scores were assessed on ICU admission. Acute respiratory distress syndrome (ARDS) was assessed according to the Berlin definition [7]. Outcome was defined as 28-day ICU mortality. 

25(OH)D was measured in serum of COVID-19 critically ill patients on ICU admission, using the electrochemiluminescence immunoassay (ECLIA) method on a Cobas E602 immunoassay analyzer (Roche Diagnostics International, Ltd., Basel, Switzerland). The vitamin D total assay employs vitamin D binding protein (VDBP) as the capture protein to bind 25-hydroxyvitamin D3 (25(OH)D_3_) and 25-hydroxyvitamin D2 (25(OH)D_2_), with a measuring range 3–70 ng/mL (defined by the limit of detection and the maximum of the master curve). The repeatability and intermediate precision coefficients of the assay were CV <5.5% and <7.0%, respectively. 

Data were expressed as mean ± standard deviation (SD) for normally distributed variables, median with interquartile range (IQR) for variables with skewed distribution, and as count (%) when categorical. Two-group comparisons were performed using the Student’s t-test, the non-parametric Mann–Whitney test, or the chi-square test, as appropriate. The Kaplan–Meier method was used for 28-day ICU mortality absence probability estimation and the log-rank test for two-group comparison. All aforementioned analyses were performed using the IBM SPSS statistics version 20 (IBM, Armonk, NY, USA). All *p* values are two-sided; *p* < 0.05 was considered statistically significant.

## 3. Results

Thirty adult patients (80% male) with a mean age of 65 years were included in the final study population. Demographics and important clinical data are given in Table 1. The patients presented with symptoms 6 days prior to ICU admission. Hypertension, hyperlipidemia and diabetes were the most common comorbidities. ARDS was present in 27 (90%) patients, mostly moderate or mild [7]. In three patients, PaO_2_/FiO_2_ was over 300 mmHg, despite the presence of diffuse infiltrates in the chest X-rays. Twenty-three (77%) patients required mechanical ventilation, and 5 died within 28 days after ICU admission.

In our cohort of critically ill COVID-19 pneumonia patients, six (20%) patients were vitamin D insufficient (20–29.9 ng/mL), and the remaining 80% were deficient (<19.9 ng/mL); no patient exhibited vitamin D sufficiency (>30 ng/mL). Therefore, given the high rate of vitamin D deficiency in our patient cohort and the limited sample size, we classified the patients according to the median of the whole cohort (low, <15.2 ng/mL, N = 15) and patients with 25(OH)D levels ≥15.2 ng/mL (high, N = 15). Most experts agree that anyone with a 25(OH)D level of less than 15 ng/mL needs more vitamin D; indeed, the Institute of Medicine has suggested that levels of 16 ng/mL meet the needs of approximately half the population [8]. Demographics, clinical and biochemical characteristics on ICU admission and important outcomes of the two patient groups are listed in Table 2. This table serves for descriptive purposes only. We note that potential confounders of differences observed are not accounted for. The two groups differed only with respect to 28-day mortality. Overall ICU mortality in the two groups did not differ; however, vitamin D-low patients died within 20 ± 7 days, whereas vitamin D-high patients within 44 ± 7 days, from ICU admission (*p* = 0.001). 

**Figure 1:**
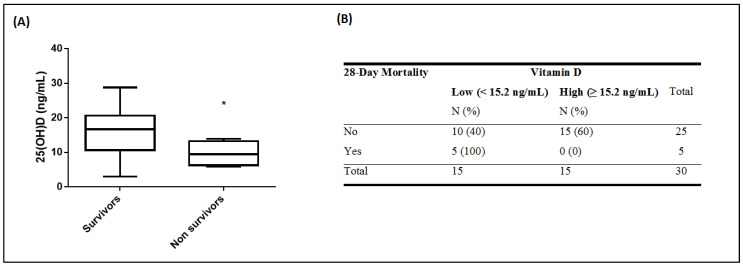
25-hydroxyvitamin D and 28-day mortality. 25(OH)D levels were measured in 30 critically ill COVID-19 patients on ICU admission. Patients were subdivided in patients who died within 28 days of ICU stay (N = 5, non-survivors), and patients who survived (N = 25, survivors). 25(OH)D levels on ICU admission were compared between the two groups. A difference is observed in 25(OH)D levels on ICU admission (A: vitamin D continuous, *p* = 0.03; B: vitamin D categorical, *p* = 0.02). 25(OH)D levels in the patients were quantified in blood samples harvested on ICU admission (within the first 48 h). Two-group comparisons were performed using the non-parametric Mann–Whitney test for skewed data for continuous vitamin D variable and chi square test for categorical vitamin D variable with the two categories (cut-off = 15.2 ng/mL). Asterisk symbol (*) means *p* < 0.05. Line in the box: median value; box edges: 25th to 75th centiles; whiskers: range of values (**A**). Data are expressed as the number of patients (N) and percentages of totals (%) (**B**).

Median 25(OH)D levels of the survivors were significantly higher than those of the non-survivors. For the survivors, the median was 16.7 ng/mL (interquartile range (IQR) of 10.6–20.7), whereas at the time of ICU admission, median 25(OH)D levels for the non-survivors were 9.4 ng/mL (IQR of 6.2–13.2; *p* = 0.03; Figure 1A). Hence, both continuous and categorical 25(OH)D levels on ICU admission differed between survivors (N = 25) and non-survivors (N = 5). Figure 1 depicts the difference observed in 25(OH)D levels on ICU admission (continuous; *p* = 0.03; Figure 1A and categorical; *p* = 0.02; Figure 1B). As seen in Figure 1B, all patients who died belonged to the low vitamin D group.

**Figure 2:**
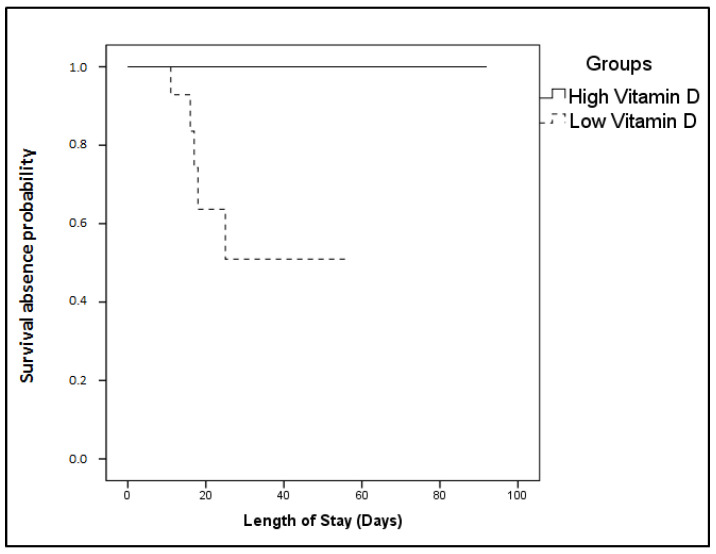
25-hydroxyvitamin D on ICU admission and 28-day ICU mortality probability. Probability for 28-day mortality based on ICU admission 25(OH)D levels. The patient cohort was dichotomized above and below the median 25(OH)D value (15.2 ng/mL). Solid line: ≥15.2 ng/mL; dashed line: <15.2 ng/mL. The Kaplan–Meier method was used for 28-day ICU mortality probability estimation and the log-rank test for two group comparison. The probability of survival absence with time was significantly elevated in the low vitamin D group (*p* = 0.01). All patients belonging to the high group survived, while all non-survivors belonged to the low group.

Finally, the Kaplan–Meier method was performed for 28-day survival absence probability estimation. The ICU cohort was independently dichotomized above and below the vitamin D median: a high vitamin D group (≥15.2 ng/mL) and a low vitamin D group (<15.2 ng/mL). The probability of survival absence with time was significantly elevated in the low vitamin D group (log-rank test, *p* = 0.01; Figure 2). All patients belonging to the high group survived, while all non-survivors belonged to the low group. A Cox survival analysis was also performed; no parameter was identified as an independent predictor of survival.

## 4. Discussion

In this observational, single center study, we demonstrated that COVID-19 non-survivors had lower ICU admission 25(OH)D levels compared to survivors, implying a possible association of low 25(OH)D levels with poor prognosis of COVID-19 pneumonia patients. 

To the best of our knowledge, this is the first, albeit, pilot study in ICU patients showing an association between 25(OH)D levels and 28-day mortality. In another pilot study with a similar number of patients, low serum levels of vitamin C and 25(OH)D were found in most of the critically ill COVID-19 ICU patients. However, older age and low vitamin C levels appeared to be co-dependent risk factors for mortality [9]. A study performed in a respiratory intermediate care unit (RICU), showed similar results to ours, i.e., survival analysis pointed out that, after 10 days of hospitalization, severe vitamin D deficiency patients (<10 ng/mL) had a 50% mortality probability [10]. A very recent study demonstrated that 25(OH)D levels are very low in critically ill COVID-19 patients, and that the corresponding inflammatory response and fatality rates are higher compared to asymptomatic carriers [11]. 

The remaining studies focused on patients hospitalized in wards. More specifically, vitamin D deficiency has been associated with the progression and severity of COVID-19, such as higher risk of invasive mechanical ventilation and/or death [12,13,14,15,16,17]. Thus, it has been stipulated that diagnosis of vitamin D deficiency could be helpful in assessing patients’ potential of developing severe COVID-19, defined as presence of ARDS and/or mechanical ventilation, ICU admission versus ward admission and lower probability of survival. In the elderly, patients with low 25(OH)D levels exhibited elevated cytokine storm markers, resulting in hypoxia that required non-invasive ventilatory support. The study, however, was underpowered to detect a significant difference in mortality [18]. Bolus 25(OH)D supplementation during or just before COVID-19 was associated in frail elderly with less severe COVID-19 and better survival rate [19,20]. In the general population, it has been suggested that low plasma 25(OH)D levels constitute an independent risk factor for COVID-19 infection and hospitalization [21], while significantly lower 25(OH)D levels have been observed in SARS-CoV2-positive patients compared with negative patients [22].

Complex associations and interactions, along with a variety of risk factors for low 25(OH)D levels, make it difficult to prove cause and effect of 25(OH)D on the outcomes of COVID-19 ICU patients. Exploratory studies with carefully chosen matched control groups are of outmost importance.

The limitations of our study should be stated. This pilot study concerned a Greek-only ethnicity population of a limited size in a single ICU. Potential confounders on low 25(OH)D levels were not accounted for. We also had one single 25(OH)D value on ICU admission; we did not have 25(OH)D measurements prior to ICU admission or serial measurements during ICU stay. We were able, however, to demonstrate that 28-day ICU non-survivors had lower continuous and categorical 25(OH)D admission levels than survivors.

## Figures and Tables

**Table 1:** Demographic, clinical characteristics and important outcomes of patients.

Parameters	All Patients
Number of patients, N (%)	30
Age (years), (mean ± SD)	65 ± 11
Sex, N (%)	
Male	24 (80)
Female	6 (20)
Sick days prior to ICU admission	6 ± 2
Comorbidities	23 (77)
Hypertension	15
Hyperlipidaemia	9
Diabetes	5
CAD	4
COPD	1
Asthma	1
Smoking	3 (10)
ARDS	27 (90)
Mild (200–300 mmHg)	11
Moderate (100–200 mmHg)	14
Severe (<100 mmHg)	2
PaO_2_/FiO_2_ (mmHg), (median, IQR)	189 (125–260)
APACHE II, (mean ± SD)	14 ± 5
SOFA, (mean ± SD)	7 ± 3
Temperature (°C), (mean ± SD)	37.5 ± 1.1
Heart rate (bpm), (median, IQR)	86 (80–104)
Mean arterial pressure (mmHg), (mean ± SD)	83 ± 15
Respiratory rate (breaths/min), (mean ± SD)	23 ± 4
White blood cell count (cells/µL), (mean ± SD)	10,000 ± 5000
Neutrophil count (cells/µL), (mean ± SD)	8000 ± 5000
Platelets (cells/µL), (median, IQR)	220,000 (180,000–280,000)
25(OH)D (ng/mL), (median, IQR)	15.2 (9.6–19.1)
CRP (mg/dL), (median, IQR)	12 (5–20)
Lactate (mmol/L), (mean ± SD)	1.3 ± 0.5
LDH (U/L), (median, IQR)	440 (350–630)
Albumin (g/dL), (mean ± SD)	3.4 ± 0.6
Globulin (g/dL), (mean ± SD)	2.8 ± 0.5
INR, (median, IQR)	1.1 (1.02–1.12)
Fibrinogen (mg/dL), (mean ± SD)	610 ± 170
CK (IU/L), (median, IQR)	150 (70–370)
CKMB (U/L), (median, IQR)	24 (17–37)
High-sensitive troponin T (ng/mL),	
(median, IQR)	20 (10–60)
Creatinine (mg/mL), (mean ± SD)	1 ± 0.3
Urea (mg/dL), (median, IQR)	45 (27–56)
Glucose (mg/dL), (median, IQR)	140 (110–190)
Total Bilirubin (mg/dL),	
(median, IQR)	0.6 (0.4–1.0)
ALT (IU/L), (median, IQR)	40 (20–60)
AST (IU/L), (median, IQR)	40 (36–62)
ALP (U/L), (median, IQR)	65 (46–88)
Amylase (U/L), (median, IQR)	66 (47–114)
γ-GT (IU/L), (media, IQR)	55 (22–78)
Outcomes	
ICU stay (days), (median, IQR)	19 (13–40)
28-Day Mortality	5 (17)
Mechanical ventilation, N (%)	23 (77)
Anti-COVID-19 therapy	30 (100)
Azithromycin/chloroquine/lopinavir/ritonavir	12
Azithromycin/chloroquine	9
Lopinavir/ritonavir/chloroquine	4
Chloroquine	2
Convalescent Plasma	2
Other	1

Data are expressed either as number of patients (N) and percentages of totals (%), mean ± SD, or median (IQR), as appropriate. All values were estimated within the first 48 h post ICU admission in critically ill patients. 25(OH)D = 25-hydroxyvitamin D; γ-GT = γ-glutamyl transpeptidase; ALP = alkaline phosphatase; ALT = alanine transaminase; APACHE = acute physiology and chronic health evaluation; ARDS = acute respiratory distress syndrome; AST = aspartate transaminase; CAD = coronary artery disease; CK = creatine kinase; CKMB = creatine kinase myocardial band; COPD = chronic obstructive pulmonary disease; CRP = C-reactive protein; ICU = intensive care unit; INR = international normalized ratio; LDH = lactate dehydrogenase; SOFA = sequential organ failure assessment.

**Table 2:** Patient characteristics on ICU admission and important outcomes stratified by 25-hydroxyvitamin D levels.

Parameters	Vitamin D		*p*-Value
	**Low**	**High**	
	<15.2 ng/mL	≥15.2 ng/mL	
Number of patients, N (%)	15 (50)	15 (50)	
Age (years), (mean ± SD)	67 ± 13	63 ± 9	0.3
Body mass index (kg/m^2^), (mean ± SD)	26.4 ± 1.9	27.6 ± 1.9	0.1
Sex, N (%)			0.4
Male	11 (73)	13 (87)	
Female	4 (27)	2 (13)	
Days sick prior to admission	6 ± 3	6 ± 2	0.7
Characteristics on ICU admission			
Comorbidities	11 (73)	12 (80)	0.7
ARDS	14 (93)	13 (87)	0.5
Mild (200–300 mmHg)	4	7	
Moderate (100–200 mmHg)	9	5	
Severe (<100 mmHg)	1	1	
PaO_2_/FiO_2_ (mmHg), (mean ± SD)	180 ± 70	210 ± 110	0.4
APACHE II score, (mean ± SD)	14 ± 5	15 ± 5	0.8
SOFA score, (mean ± SD)	7 ± 3	7 ± 3	0.5
Temperature (°C), (mean ± SD)	37.6 ± 1.0	37.4 ± 1.1	0.6
Heart rate (bpm), (median, IQR)	85 (78–105)	88 (82–100)	0.4
Mean arterial pressure (mmHg), (mean ± SD)	85 ± 19	82 ± 11	0.6
Respiratory rate (breaths/min),			
(mean ± SD)	24 ± 5	22 ± 3	0.2
White blood cell count (cells/µL), (mean ± SD)	11,000 ± 5000	10,000 ± 5000	0.3
Neutrophils (cells/µL), (median, IQR)	7000 (5000–14,000)	5000 (3000–7000)	0.2
Platelets (cells/µL), (median, IQR)	220,000 (190,000–370,000)	220,000 (140,000–250,000)	0.2
CRP (mg/dL), (median, IQR)	19 (5–26)	10 (4–17)	0.06
Lactate (mmol/L), (mean ± SD)	1.3 ± 0.5	1.2 ± 0.6	0.7
LDH (U/L), (median, IQR)	470 (400–640)	420 (340–530)	0.9
Albumin (g/dL), (mean ± SD)	3.3 ± 0.6	3.5 ± 0.5	0.2
Globulin (g/dL), (mean ± SD)	2.9 ± 0.5	2.7 ± 0.5	0.2
INR, (median, IQR)	1.11 (1.05–1.30)	1.05 (1.00–1.10)	0.1
Fibrinogen (mg/dL), (mean ± SD)	660 ± 190	570 ± 140	0.2
CK (IU/L), (median, IQR)	200 (70–300)	120 (80–510)	0.2
CKMB (U/L), (median, IQR)	23 (18–42)	29 (17–37)	0.3
High-sensitive troponin T (ng/mL), (median, IQR)			
	39 (14–125)	14 (10–27)	0.1
Creatinine (mg/mL), (mean ± SD)	1.2 ± 0.4	0.9 ± 0.3	0.1
Urea (mg/dL), (median, IQR)	48 (25–63)	31 (27–56)	0.4
Glucose (mg/dL), (median, IQR)	130 (117–182)	170 (109–196)	0.7
Total Bilirubin (mg/dL),			
(median, IQR)	0.7 (0.5–0.9)	0.6 (0.4–1.0)	0.9
ALT (IU/L), (median, IQR)	44 (20–70)	38 (23–49)	0.3
AST (IU/L), (median, IQR)	46 (33–63)	41 (37–61)	0.5
ALP (U/L), (median, IQR)	68 (50–80)	55 (41–117)	0.9
Amylase (U/L), (median, IQR)	64 (40–93)	75 (55–132)	0.8
γ-GT (IU/L), (media, IQR)	43 (20–70)	60 (24–89)	0.9
Outcomes			
28-Day ICU mortality, N (%)			0.01 *
No	10 (67)	15 (100)	
Yes	5 (33)	0 (0)	
Day of ICU death (days), (mean ± SD)	20 ± 7	44 ± 7	0.001 *
ICU stay (days), (median, IQR)	17 (13–30)	35 (11–54)	0.1
Mechanical ventilation, N (%)	11 (73)	12 (80)	0.7

* *p* < 0.05. Patients were categorized according to their 25(OH)D levels on admission; the two patient groups were those with lower than the median 25(OH)D levels (low, <15.2 ng/mL) and those with higher than the median (high, ≥15.2 ng/mL). Data are expressed either as number of patients (N) and percentages of totals (%), mean ± SD, or median (IQR), as appropriate. Two-group comparisons were performed using the Student’s *t*-test or the non-parametric Mann–Whitney test for skewed data. Associations between qualitative variables were examined by the chi-square test. All characteristics were estimated within the first 48 h post ICU admission. γ-GT = γ-glutamyl transpeptidase; ALP = alkaline phosphatase; ALT = alanine transaminase; APACHE = acute physiology and chronic health evaluation; ARDS = acute respiratory distress syndrome; AST = aspartate transaminase; CK = creatine kinase; CKMB = creatine kinase myocardial band; CRP = C-reactive protein; ICU = intensive care unit; INR = international normalized ratio; LDH = lactate dehydrogenase; SOFA = sequential organ failure assessment.
